# Probing the localization of magnetic dichroism by atomic-size astigmatic and vortex electron beams

**DOI:** 10.1038/s41598-018-22234-8

**Published:** 2018-03-05

**Authors:** Devendra Singh Negi, Juan Carlos Idrobo, Ján Rusz

**Affiliations:** 1 Department of Physics and Astronomy, Uppsala University, Uppsala, 75237 Sweden; 2 Oak Ridge National Laboratory, Center for Nanophase Materials Sciences, Oak Ridge, Tennessee 37831 USA

## Abstract

We report localization of a magnetic dichroic signal on atomic columns in electron magnetic circular dichroism (EMCD), probed by beam distorted by four-fold astigmatism and electron vortex beam. With astigmatic probe, magnetic signal to noise ratio can be enhanced by blocking the intensity from the central part of probe. However, the simulations show that for atomic resolution magnetic measurements, vortex beam is a more effective probe, with much higher magnetic signal to noise ratio. For all considered beam shapes, the optimal SNR constrains the signal detection at low collection angles of approximately 6–8 mrad. Irrespective of the material thickness, the magnetic signal remains strongly localized within the probed atomic column with vortex beam, whereas for astigmatic probes, the magnetic signal originates mostly from the nearest neighbor atomic columns. Due to excellent signal localization at probing individual atomic columns, vortex beams are predicted to be a strong candidate for studying the crystal site specific magnetic properties, magnetic properties at interfaces, or magnetism arising from individual atomic impurities.

## Introduction

Rapid growth in nanotechnology and newly emerging magnetic structures at nano and atomic scale, are simultaneously demanding for tools and techniques, capable of characterizing the electrical and magnetic properties of material at nano and atomic scale^1–11^. In this context, with substantial development in instrumental and theoretical front of electron energy-loss spectroscopy (EELS) in (scanning) transmission electron microscopy ((S)TEM); EELS offers such capabilities. In TEM and STEM, Electron Magnetic Circular Dichroism (EMCD) method using EELS is capable of nano scale and atomic resolution magnetometry^12^, respectively. EMCD is analogous to X-Ray Magnetic Circular Dichroism (XMCD) in its capability to provide the magnetic information, but with superior spatial resolution^13–25^. In principle atomic resolution magnetic information can be achieved from EMCD experiments, by using electron vortex beams and four fold astigmated probes in STEM^26–34^. Producing electron vortex beams in STEM requires some instrumental modification, while phase aberrated probes can be shaped by utilizing the optics of an aberration corrector, which are now available in most modern state-of-art electron microscopes. In a recent report, it was argued that the phase in the tail of aberrated beams behaves analogously as polarization in light or X-rays. This allows to probe the asymmetry of dichroic signals at atomic scale using the phase of aberrated beams^32^. Electron vortex beams have also emerged as a promising candidate for atomic scale magnetometry^35–39^.

Detection of magnetism in principle can be reached at the atomic level by using atomic size electron probes. However, due to the intrinsic delocalization of the magnetic signal, there still exists a non-negligible probability that a magnetic electronic excitation to be detected even if occurs away from the probe, at a distance of the order of few Ångströms. That means that, in addition to the probed atomic column, the magnetic signal can originate from neighboring atomic columns. Moreover, the electron probe also expands as it propagates through the crystal, resulting in the detection of a magnetic signal from more distant atomic columns. In order to rationalize the spatial resolution that can achived in EMCD, it is important to understand the source and localization of the magnetic signal strength^40–46^.

Recently, localization of the EMCD signal has been studied in the experimental geometries with tilted crystal, particularly in a three-beam geometry^43^. There, it was found that EMCD is strongly localized within the probed atomic plane, while the non-magnetic EELS signal presented a stronger delocalization than EMCD. However, tilting the crystal to three-beam orientation greatly reduces the dynamical diffraction effects, resulting in simultaneously loosing the capability of probing individual atomic columns.

Here, we study the localization of the magnetic dichroic signal on single atomic columns using customized phase aberrated probes and vortex beams. The material chosen for this study is an antiferromagnet LaMnAsO. LaMnAsO is an ideal testing system due to its interesting physical properties, antiferromagnetic ordering and a broad range of atomic masses leading to non-trivial dynamical diffraction effects. The electron beam was set parallel to the crystal *c*-axis, resulting in a stronger dynamical diffraction effects when compared to a three-beam orientation geometry. The optimized collection angle for all considered types of beam shapes is defined for a calculated EMCD with the highest signal to noise ratio (SNR). In addition, the magnetic and nonmagnetic excitations were calculated from the probed and neighboring atomic columns as a function of material thickness to analyze the spatial origins of the EMCD signal.

## Methods

**Figure 1:**
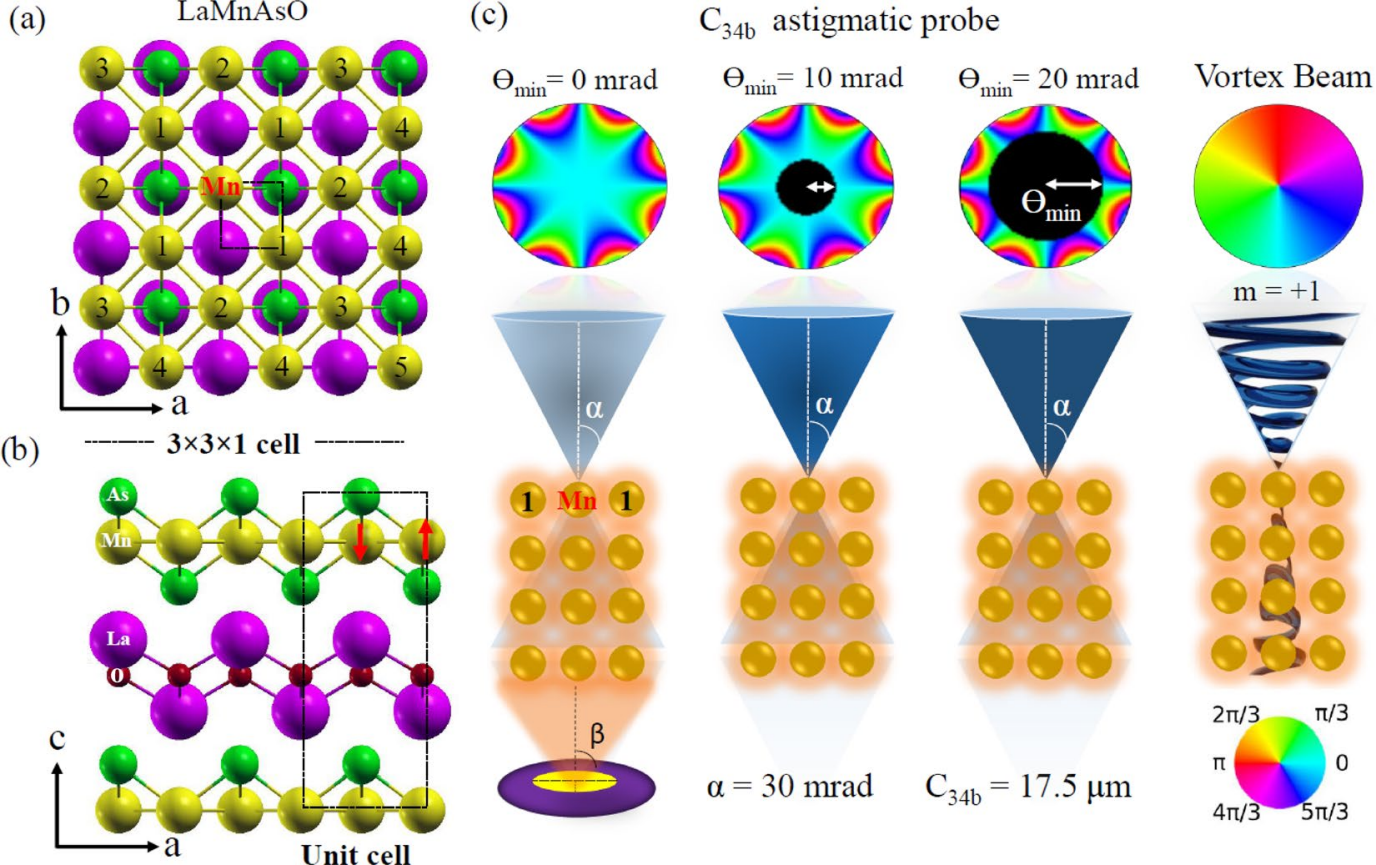
(**a**,**b**) Projectons of the LaMnAsO crystal structure and (**c**) various probes used to study the atomic signal delocalization. The numbers in panel (a) denote the nearest neighbor groups of Mn atomic columns relative to the column marked “Mn”, where the electron beam was positioned.

The atomic scale localization of the dichroic signal is studied using LaMnAsO as a test bed. LaMnAsO belongs to the 1111 family of iron based superconductors with layered crystal structure^47^. The schematic of the crystal structure of LaMnAsO, is shown in Fig. 1. LaMnAsO belongs to the space group P4/nmn, with lattice parameters of *a* = 0.412 nm, *c* = 0.903 nm^48^. In LaMnAsO, Mn has a magnetic moment of ~2.4 *μ*_*B*_ at 300 K. Magnetic moments of Mn are aligned ferromagnetically along the *c* axis, while in the *ab* plane they order antiferromagnetically in a checkerboard pattern.

Previously, in LaMnAsO the magnetic ordering was studied theoretically and experimentally using a four-fold astigmatic probe^34^. In the present study, the magnetic dichroic signal localization is studied by simulating the energy filtered diffraction pattern of the Mn-*L*_3_ edge excitation. Inelastic scattering cross-sections are calculated with combined a multislice/Bloch waves approach^29^, as implemented in the MATS.v2 software^49^. [3 × 3 × 1] unit cell of LaMnAsO with a grid spacing of 6.4 pm, is used for the multislice calculation. The unit cell is aligned along the [001] zone axis. An optimized value of four fold astigmatism *C*_34*b*_(17.5 *μ*m) and convergence angle *α* = 30 mrad was considered for all the phase aberrated probe calculations^33^. The energy filtered mixed dynamic form factor (MDFF) matrix elements are calculated with sum rule inversion method, under the dipole approximation^20,44^. Scattering angles are chosen up to ±40 mrad in both *θ*_*x*_, *θ*_*y*_ direction. All the calculations were performed with probes accelerated at 100 kV, within the z-locality approximation^50^. The MATS.v2 summation convergence parameter^49,51^ was set to 1 × 10^−6^.

Two different types of probes – four-fold astigmatic electron beam and electron vortex beam – were used to compute the signal distribution over the atomic columns. Calculations are also carried out by using annular convergence apertures with inner angles of 10 and 20 mrad. The vortex beam used here was set with an orbital angular moment (OAM) $\langle {\hat{L}}_{z}\rangle =1\hslash$, as shown in Fig. 1. In this manuscript the inner probe-forming aperture size is denoted as Θ_min_.

Neglecting the prefactor, the double-differential scattering cross-section (DDSCS) is given by

1 $$\frac{{\partial }^{2}\sigma }{\partial E\,\partial {\rm{\Omega }}}\propto \sum _{I,F}\,|\langle {{\rm{\Psi }}}_{out}|\otimes \langle F|\hat{V}|I\rangle \otimes |{{\rm{\Psi }}}_{in}\rangle {|}^{2}\delta ({E}_{F}-{E}_{I}-E)$$

Equation 1 represents the DDSCS for energy loss *E*. |*I*〉, |*F*〉 are the initial and final state of atomic electron, with energies *E*_*I*_, *E*_*F*_, respectively. |Ψ_*out*_〉, |Ψ_*in*_〉 represent the outgoing and incoming probe wave function, respectively. The magnetic signal is obtained by calculating the mixed dynamical form factor (MDFF)^52–54^. MDFF represents interference terms of the incoming and outgoing electron probe in the cross section induced by two non-equal momentum transfer vectors. The MDFF for momentum transfer vectors **q**, **q**′ is given by

2 $$S({\bf{q}},{\bf{q}}^{\prime} ,E)=\sum _{I,F}\,\langle F|{e}^{-2\pi i{\bf{q}}.{\bf{r}}}|I\rangle \langle I|{e}^{2\pi i{\bf{q}}^{\prime} .{\bf{r}}}|F\rangle \delta ({E}_{F}-{E}_{I}-E)$$

In the dipole approximation, for a material magnetized along the *z*-axis, the MDFF of a single atom can be written in the following form

3 $$S({\bf{q}},{\bf{q}}^{\prime} ,E)={\bf{q}}\cdot {\mathbb{N}}(E)\cdot {\bf{q}}^{\prime} +i{({\bf{q}}\times {\bf{q}}^{\prime} )}_{z}{M}_{z}(E)$$

where ${\mathbb{N}}(E)$ is a real 3 × 3 symmetric energy-loss-dependent tensor describing the non-magnetic part of the local electronic structure and *M*_*z*_(*E*) is the magnetic part. Equation 3 neglects non-dipole transitions, importance of which increases with the scattering angles. However, EMCD effect stems from dipole transitions and as will be seen below, optimal measurement conditions are obtained at small collection angles, where dipole transitions are dominating. Therefore the dipole approximation used here should provide sufficiently accurate predictions. For an orthogonal cell with crystal axes aligned parallel with *x*, *y*, *z* coordinates, the tensor ${\mathbb{N}}(E)$ becomes diagonal and for a tetragonal cell the following relation holds: *N*_*xx*_(*E*) = *N*_*yy*_(*E*) ≠ *N*_*zz*_(*E*). Thus for LaMnAsO we can write

4 $$S({\bf{q}},{\bf{q}}^{\prime} ,E)=({q}_{x}{q^{\prime} }_{x}+{q}_{y}{q^{\prime} }_{y}){N}_{xx}(E)+{q}_{z}{q^{\prime} }_{z}{N}_{zz}(E)+i({q}_{x}{q^{\prime} }_{y}-{q}_{y}{q^{\prime} }_{x}){M}_{z}(E)$$

**Figure 2:**
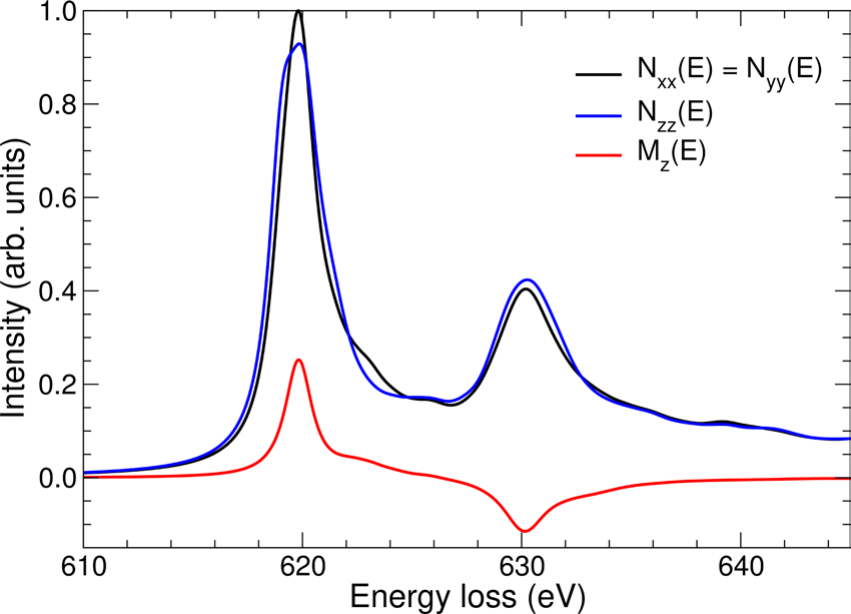
Non-magnetic, *N*_*xx*_(*E*), *N*_*yy*_(*E*), *N*_*zz*_(*E*), and magnetic, *M*_*z*_(*E*), spectral components of mixed dynamical form factors calculated by density functional theory for LaMnAsO in the ground state.

Figure 2 shows the spectral components *N*_*xx*_(*E*), *N*_*zz*_(*E*) and *M*_*z*_(*E*) as evaluated by density functional theory in the ground state (WIEN2k code^55^ using generalized gradient approximation^56^) using experimental values of the structure parameters of LaMnAsO. As can be seen, despite the elongated tetragonal unit cell, the spectral shape – as reflected by *N*_*xx*_(*E*) vs *N*_*zz*_(*E*) – is nearly isotropic. Neglecting the small anisotropy introduce a small error in the calculations, more specifically, less than 3% in the estimation of the non-magnetic spectral component of the scattering cross-section. However, assumming an isotropic condition allows to reduce the number of free parameters in the calcuations and express the MDFF as

5 $$S({\bf{q}},{\bf{q}}^{\prime} ,E)\approx {\bf{q}}\cdot {\bf{q}}^{\prime} N(E)+i({q}_{x}{q^{\prime} }_{y}-{q}_{y}{q^{\prime} }_{x}){M}_{z}(E)$$

where

6 $$N(E)=\frac{1}{3}({N}_{xx}(E)+{N}_{yy}(E)+{N}_{zz}(E)).$$

The calculated signal to noise ratio (SNR) in this study was defined as follows. The signal was obtained from the integrated magnetic spectral component of the Mn-*L*_3_ edge, and the noise as the integrated non-magnetic spectrum within the same energy range^43^. Assuming that the dynamical diffraction conditions will depend negligibly on the precise value of energy-loss within the range of Mn-*L*_3_ edge (640–650 eV), one can calculate separately a normalized scattering cross-section originating from a non-magnetic part of the electronic structure, *σ*_*nm*_, by setting

7 $${S}_{nm}({\bf{q}},{\bf{q}}^{\prime} ,E)={\bf{q}}\cdot {\bf{q}}^{\prime} ,$$

and a normalized magnetic part of the scattering cross-section, *σ*_*mag*_, using

8 $${S}_{mag}({\bf{q}},{\bf{q}}^{\prime} ,E)=i({q}_{x}{q^{\prime} }_{y}-{q}_{y}{q^{\prime} }_{x}),$$

resulting in *S*_*nm*_ and *S*_*mag*_ independent of the electronic structure. If one wishes to convert the normalized values of the non-magnetic and magnetic components of the scattering cross-section to EEL spectra, one needs to multiply them by *N*(*E*) and *M*_*z*_(*E*), respectively. Eventually, for a SNR analysis the multiplication is done by energy-integrated *N*(*E*) and *M*_*z*_(*E*), over the Mn-*L*_3_ edge

9 $$N={\int }_{{L}_{3}}\,N(E)dE\quad {\rm{and}}\quad M={\int }_{{L}_{3}}\,{M}_{z}(E)dE,$$

though, these values enter only as a scaling factor *M*/*N* in the expression for the SNR^33^

10 $$SNR={f}_{red}\frac{M}{N}\frac{{\sigma }_{mag}}{{\sigma }_{nm}}\sqrt{2\frac{{N}_{pix}{C}_{{L}_{3}}}{1+b}}$$

where *f*_*red*_ is an estimated overall reduction of the EMCD signal originating from summation over pixels adjacent to the center of an atomic column. *σ*_*nm*_, *σ*_*mag*_ introduced above are scattering cross-sections calculated using Eqns. 7 and 8, respectively. *N*_*pix*_ is the number of pixels around the atomic column from which the signal is accumulated, ${C}_{{L}_{3}}$ is sum of inelastically scattered electrons detected within the *L*_3_ edge energy range (excluding power-law background) and *b* is the ratio of electron counts in the power-law background vs edge signal ${C}_{{L}_{3}}$. Factor of two inside the square root originates from the way, how EMCD signal is extracted – as a difference of two spectra. This doubles the magnetic signal, while the noise increases only by approximately a factor of $\sqrt{2}$.

In summary, for SNR optimization purposes, it is sufficient to work with *S*_*nm*_ and *S*_*mag*_. In plots presented below, the SNRs are normalized per unit of beam current hitting the sample, and are expressed in arbitrary units. To convert the optimized SNR to realistic values, one can use for instance parameters *f*_*red*_ = 0.8, *N*_*pix*_ = 3 × 3, ${C}_{{L}_{3}}=1000$ and *b* = 2, as in the above-mentioned paper^33^, and *M*/*N* originating from DFT calculations is approximately 0.15 within the Mn-*L*_3_ edge region. Then *SNR* ≈ 9.3*σ*_*mag*_/*σ*_*nm*_ per atomic column, assuming that the above-mentioned parameter values can be reached in the measurement using different electron microscopes.

## Results and Discussion

### Elastic probe propagation: spreading and distortion

**Figure 3:**
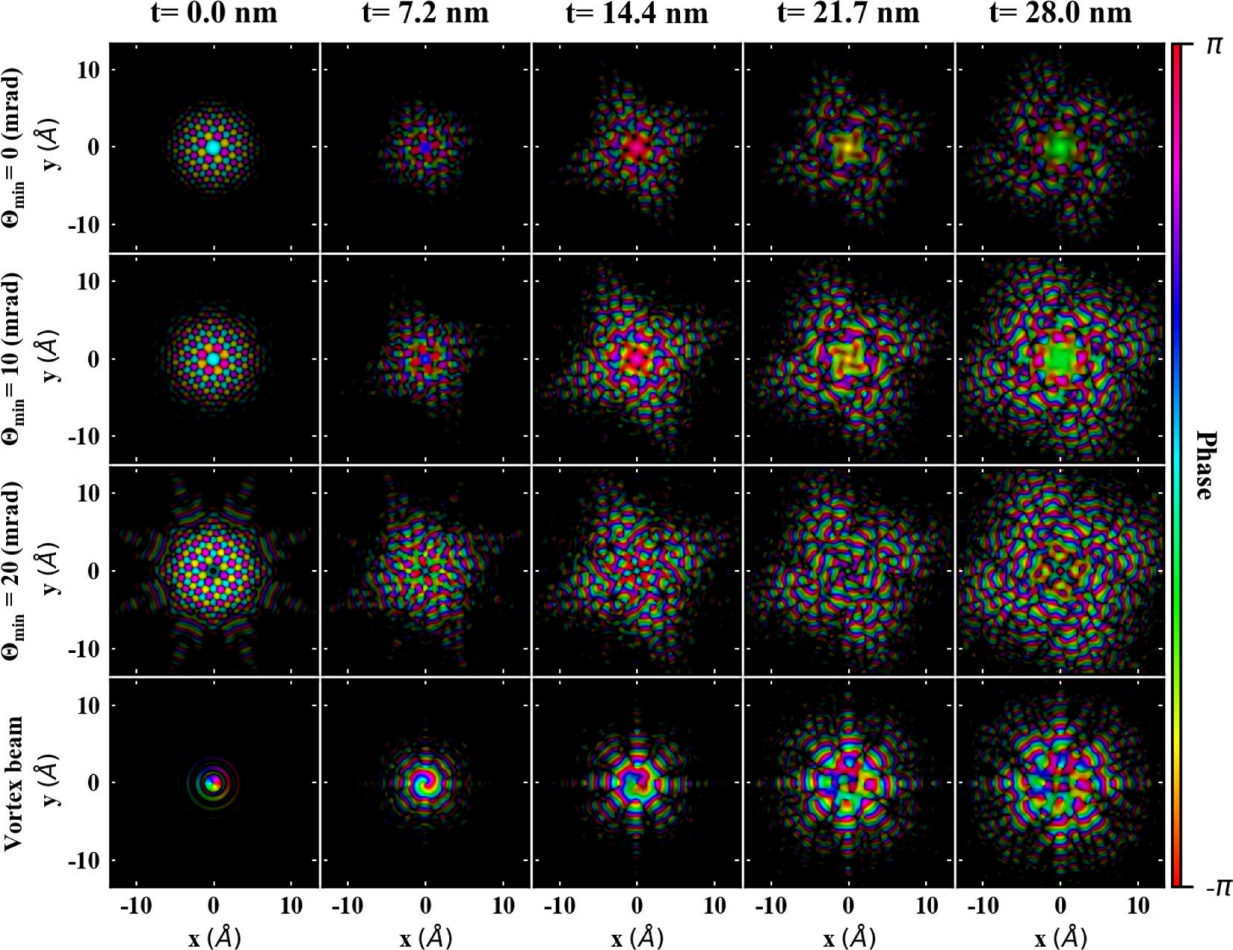
Wave function of all probes at various thicknesses in real space. Probe is focused on “Mn” site. Θ_min_ represent the aperture size in miliradian to restrict the intensity in the center portion of probe. Amplitude is shown as the saturation of the color, while phase is shown by hue (see colormap).

Figure 3 visualizes the spreading and distortion on the amplitude and phase of the probes during their elastic propagation through the crystal. Understanding of probe spreading is an important factor for studying sources of inelastic scattering signal, because a broader probe can more easily lead to an excitation of distanced atomic columns. Moreover, the phase distortion indicates the thickness range, where the probe retains its intrinsic nature. A detailed description of the shape and flux distribution of the probes can be found in the supplementary material.

At the crystal entrance plane, the aberrated probes have their maxima at the center, except for the astigmatic probe with Θ_min_ = 20 mrad, which presents enhanced tails with reduced intensity at its center. All the probes spread and distort as they propagate through the crystal. In comparison to vortex beam, aberrated beams present a stronger spread. This suggests that with aberrated probes there can be more significant contributions to the inelastic scattering cross-section from the neighboring atomic columns. However, vortex beam spreads and distorts as well, and after about 14 nm the central phase singularity and characteristic phase winding cannot be anymore recognized. Therefore one can anticipate that for larger thicknesses the efficiency of the vortex beams in detecting EMCD signal will be reduced. These qualitative expectations will be analyzed in detail in the following sections, by explicit simulations of DDSCS.

### Thickness and collection angle dependence of inelastic scattering cross-section

**Figure 4:**
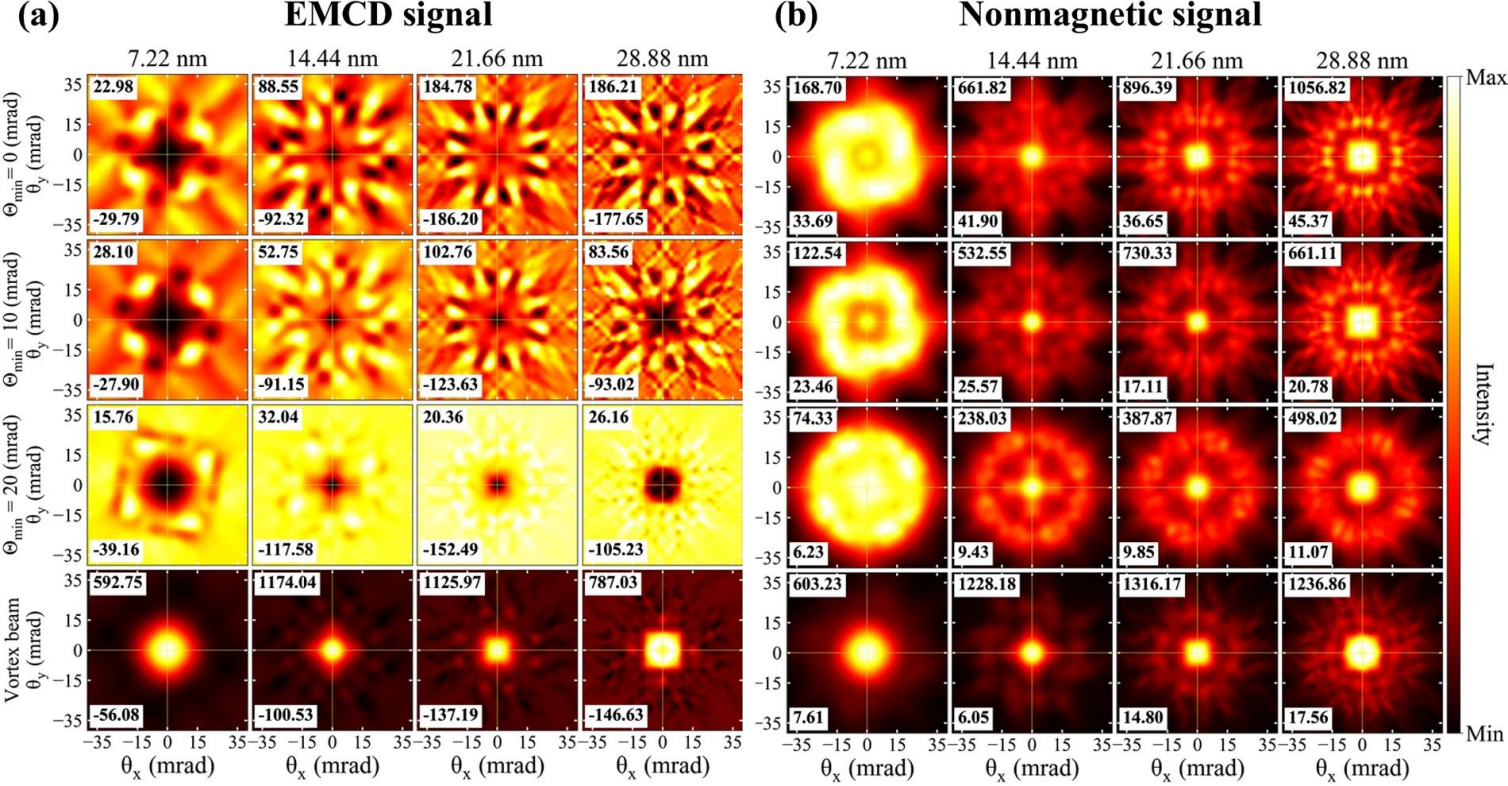
Thickness dependent (**a**) EMCD and (**b**) nonmagnetic signal for various probes. Colormap extends from minimal to maximal intensity, indicated separately in each panel (in arbitrary units).

Figure 4(a,b) show the energy filtered diffraction patterns of Mn-*L*_3_ edge for magnetic and nonmagnetic components of the DDSCS, as a function of scattering angles *θ*_*x*_, *θ*_*y*_. Each panel shows the maximum and minimum magnetic signal intensity values of the diffraction pattern. Symmetry of the four fold astigmatism is well reflected in the diffraction pattern. The dichroic signal is localized at the center for thin samples and smears out with increasing thickness. However, for the case of the aberrated probe with large inner aperture, the intensity remains within a small range of scattering angles. For aberrated probes, the maximum scattering cross section value of the magnetic signal oscillates as a function of thickness. At 7.22 nm the maximum signal intensity is present in the Θ_min_ = 20 mrad probe, whereas at 28.88 nm the maximum signal intensity is present in the Θ_min_ = 0 mrad probe. In both cases, the maxima are present at higher scattering angles. Note, however, that these maxima occur in an antisymmetric pattern with respect to the horizontal, vertical and diagonal axes, and as such they do not contribute to the total magnetic signal detected by on-axis circular detector entrance aperture (see below).

In comparison to astigmatic beams, the vortex beam shows a stronger magnetic scattering cross section localized in a small range of scattering angles. Notice that for the Θ_min_ = 20 mrad probe there are notable similarities in the distribution of the magnetic signal in the diffraction patterns when compared to the vortex beam. However, the magnetic signal has the opposite sign, result that will be explained once we analyze separately the contributions of neighboring atomic columns in the next subsection.

Figure 4(b) shows the nonmagnetic component of the scattering cross-section. For small thicknesses the nonmagnetic response arises from a wide range of scattering angles, while with increasing thickness the signal accumulates in a smaller range of scattering angles, reflecting the spreading of the beam with increasing thickness. The intensity of nonmagnetic signal oscillates with thickness. Moreover, by introducing the annular aperture Θ_min_ = 20 mrad, the maximum nonmagnetic signal intensity can be reduced up to 2 times.

**Figure 5:**
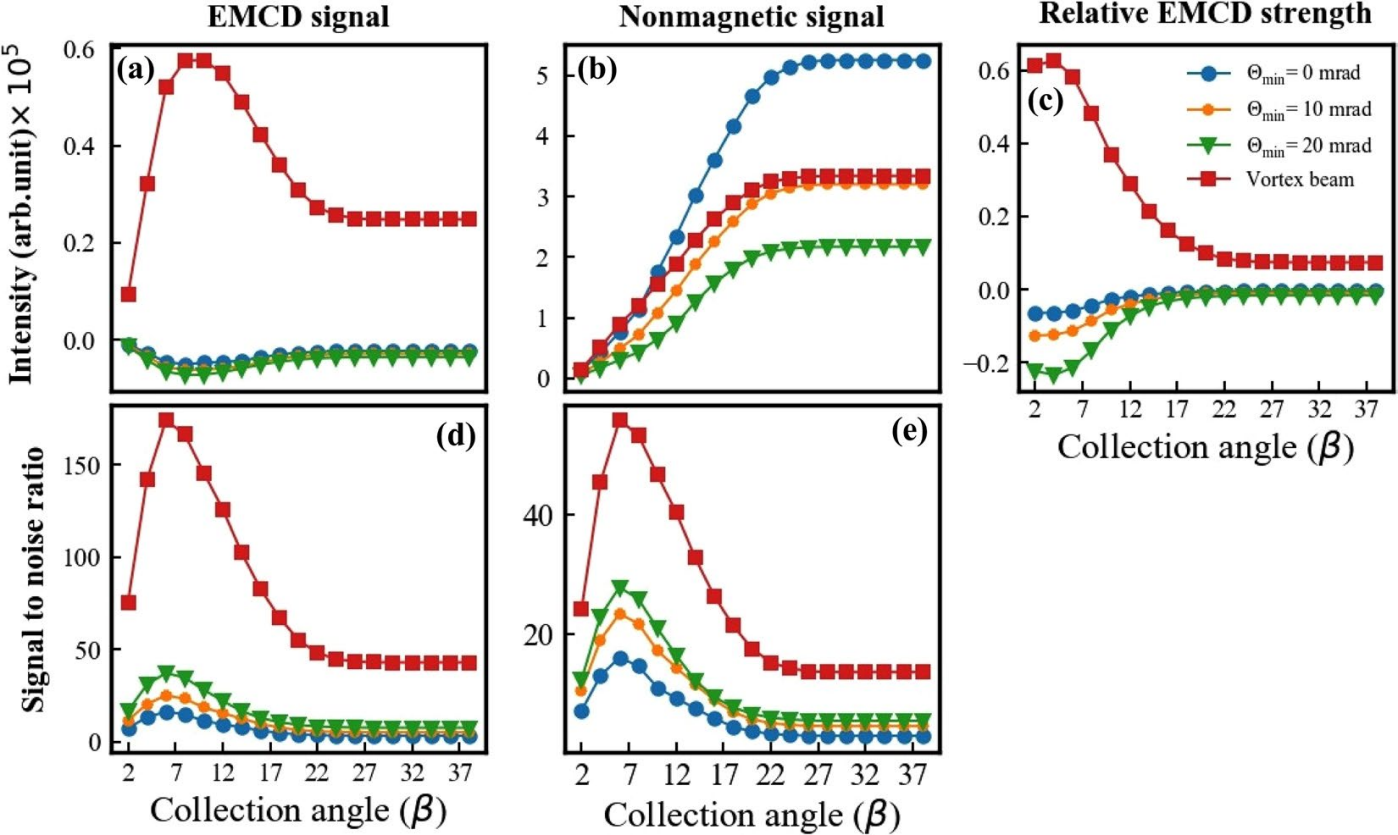
Radial profiles of (**a**) EMCD, (**b**) non-magnetic, (**c**) relative EMCD, (**d**) SNR normalized per unit of beam current hitting the sample, and (**e**) SNR normalized per unit of beam current prior to probe forming aperture (annular or fork), shown for various probes at 28.88 nm sample thickness.

In a typical STEM spectrum imaging experiment, the data is collected with an on-axis circular detector aperture. Figure 5 shows the radial profiles of magnetic signal, nonmagnetic signal, relative EMCD, SNR for the 28.88 nm thick crystal as a function of collection semi-angle. Optimized collection angles are deduced for all four considered probes. The maximum magnetic signal strength is obtained with a vortex beam within a collection angle range of 10 mrad. Per unit of beam current hitting the sample, the electron vortex beam leads to a significantly higher magnetic signal than any of the astigmatic probes. The optimal values differ by an order of magnitude, see also Figs. S1, S2 and S3 in the Supplementary information. The magnetic response of the astigmatic probes is visibly enhanced by use of the annular probe-forming aperture, yet it does not reach the performance obtained by an electron vortex beam. Note also that the local maxima in the EMCD strength seen for Θ_min_ < 20 mrad at larger scattering angles, Fig. 4(a), do not contribute to these radial profiles. As anticipated above, the observed antisymmetric magnetic distribution leads to its cancellation.

Nonmagnetic contributions necessarily increase monotonously with larger collection angles. For this reason, the optimal strength of a raw magnetic signal might not directly correspond to the optimal detection conditions due to the large nonmagnetical signal, which dominates the EEL spectra, as is discussed below. When integrated over large collection angles, the nonmagnetic signal has a maximum for the case of the astigmatic probe with Θ_min_ = 0 mrad. Larger contributions of the non cylindrical phases in the probe, see Fig. 1(c), seem to decrease the overall inelastic scattering cross-section. An astigmatic probe with Θ_min_ = 20 mrad shows the strongest reduction of the nonmagnetic signal, which improves chances for the detection of an EMCD signal.

The relative EMCD strength is maximized at very small collection semi-angles, being the highest at 4 mrad. This is not likely to be an optimal measurement condition either, because of the very small amount of detected electrons. Vortex beam clearly surpasses the other considered astigmated probes with higher values of both the relative EMCD signal and SNR. The optimal SNR is predicted to be about 5 times higher than that of the aberrated probe. Radial profiling suggests that most of the magnetic contribution is concentrated at small scattering angles, constraining the optimal magnetic signal to a relative small collection angle of ~6 mrad. This finding should further encourage attempts for the experimental verification. We observe similar trends for other thicknesses [Supplementary Figs. S1, S2 and S3].

Note that the SNR is normalized per unit of beam current hitting the sample, as was described in the Methods section. If we would consider a different normalization, per beam current in the column after passing through the annular or fork aperture, respectively, then the current hitting the sample is reduced by factors 1, 8/9, 5/9 and ~1/10^35^ for astigmatic probes with Θ_min_ = 0, 10, 20 mrad and vortex beam, respectively. This leads to a SNR reduction factors of 1, 0.94, 0.75 and 0.32. SNRs normalized this way are shown in Fig. 5(e). The SNR can be enhanced by introducing the annular convergence aperture while probing with an aberrated beam. Among the aberrated probes Θ_min_ = 20 mrad shows the highest SNR within the small window of collection angle range of (6 mrad). Θ_min_ = 0, 10 mrad shows optimal SNR in a higher collection angle range, but overall the SNR value is lower than for Θ_min_ = 20 mrad. Vortex beams still maintain their advantage over astigmatic probes, although the difference is somewhat reduced. A vortex beam shows more than 2 times better SNR when compared to the aberrated probes. All the probes shows a signal saturation at higher collection angles.

### Magnetic dichroic signal localization on atomic columns

**Figure 6:**
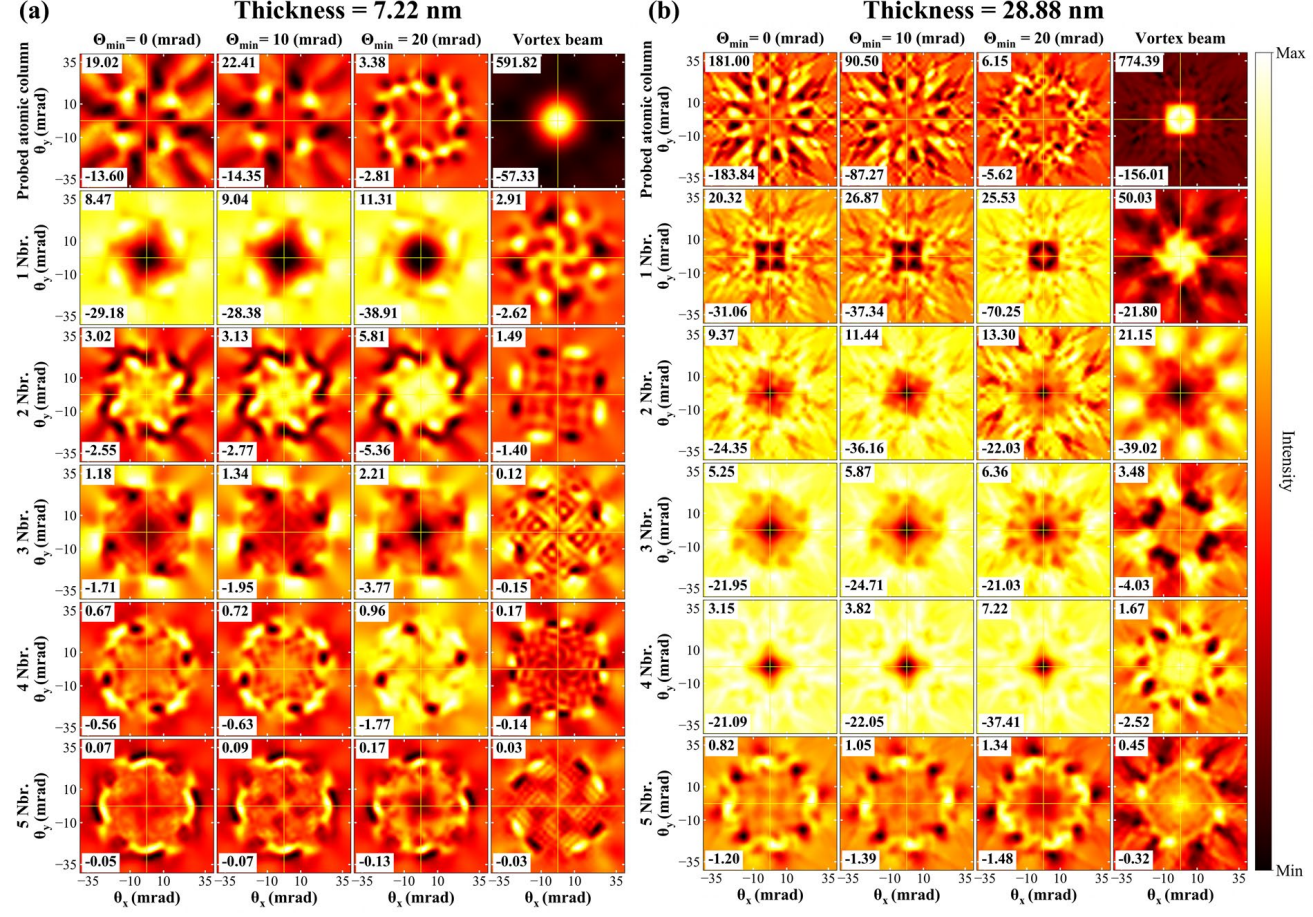
EMCD contribution from individual atomic columns for all probes at (**a**) 7.22 nm (**b**) 28.88 nm sample thicknesses.

Figure 6(a,b) show the magnetic component of energy filtered diffraction pattern of Mn-*L*_3_ edge originating from various atomic columns in the LaMnAsO crystal, see Fig. 1(a). Figure 6 represents a decomposition of the magnetic dichroic signal discussed in the previous section, into contributions from individual groups of neighboring atomic columns and as such it is an explicit visualization of the delocalization of the magnetic signal. Individual columns represent the signal originating from various types of probes, while the rows show the contribution from the probed atomic column and its neighbors atoms, following the atomic column labeling shown in Fig. 1.

An interesting observation can immediately be made for astigmatic probes. The maximum magnetic contribution comes from the nearest neighbor atoms, rather than from the probed atomic column, and gradually decays for the more distant neighbor atoms. This behavior is observed for all sample thicknesses considered here and explains the opposite sign of magnetic signal observed in Fig. 4a. The magnetic signal originating from the probed atomic column shows relatively high absolute values of an EMCD signal, but its distribution shows a tendency for a four fold symmetry. For small thicknesses, 7.22 nm (and 14.44 nm), and with Θ_min_ = 0, 10 mrad the maximal absolute values of the probed column and the nearest neighbor columns are comparable. This scenario however changes with Θ_min_ = 20 mrad, as the maximum magnetic intensity value from the probed atomic column drastically reduces to about 9% as compared to the nearest neighbor atomic columns [see also Figs. S4 and S5 in Supplementary Information]. Thus with increasing annular inner angle, Θ_min_, the intensity gradually localizes on the nearest neighbor atomic columns. For the whole thickness range, vortex beams show a consistent pattern of magnetic signal profiling. The maximum magnetic signal stems from the probed atomic column and it is drastically reduced to the distanced neighbor atomic columns. The maximum magnetic signal lies in the center of energy filtered diffraction pattern (EFDP), within a small range of scattering angles. EFDP suggests that vortex beam remains strongly localized at the probed atomic column, as compared to the aberrated beams.

Vortex beams seem to be better candidates to probe atomically localized magnetic properties of the materials. However, EFDP also indicates that vortex beams are better suitable for thin samples (within ≈ 10 nm), presumably due to strong distortion of the probe with thicker samples, see Fig. 3. Previous studies using the Bloch waves method explored the elastic dynamic scattering of electron vortex beams in crystals^57,58^. Such studies showed that with higher semi-convergence angle (30 mrad, *m* = +1), a vortex beam remains strongly localized with in few (<10) nanometers. Beyond approximately 10 nm the vortex beam spreads and a non-negligible signal contributions begins to arise from the neighboring atomic columns.

### Nonmagnetic signal localization on atomic columns

**Figure 7:**
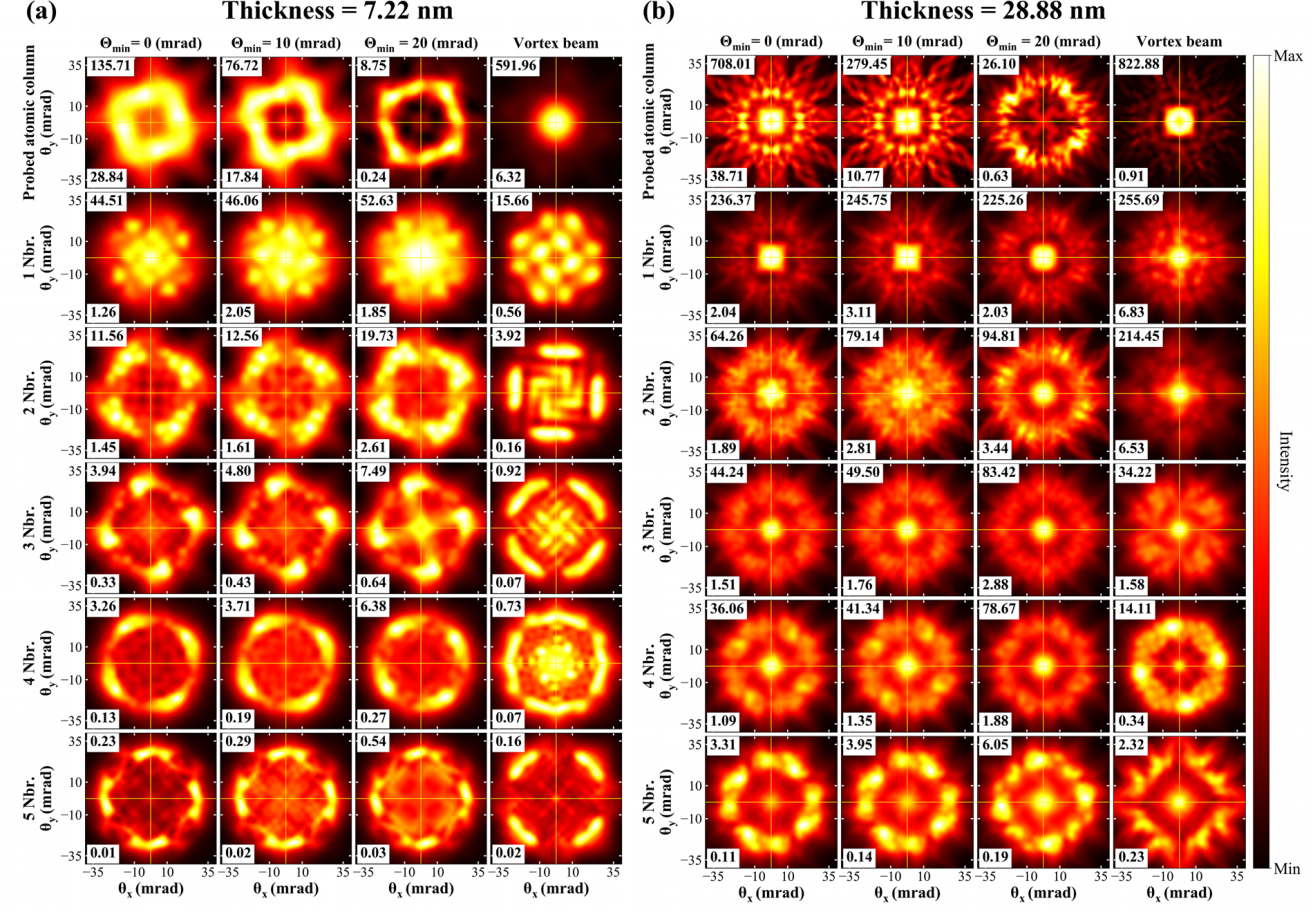
Nonmagnetic contribution from individual atomic columns for all probes at (**a**) 7.22 nm and (**b**) 28.88 nm sample thicknesses.

Figure 7(a,b) show the EFDP of Mn-*L*_3_ edge for the nonmagnetic signal component originating from individual atomic columns. Every column represents a specific probe type, and the rows represent the probed and neighboring atomic columns. Comparing the nonmagnetic signal response among the aberrated probes, the strongest signal intensity originates from the probed atomic columns for all calculated thicknesses for Θ_min_ = 0, 10 mrad probes. The non-magnetic response decays on more distant atomic columns, while the nearest atomic columns show only about one third of the intensity coming from the probed column. The larger signal intensities are limited to a relatively small range of scattering angles.

The Θ_min_ = 20 mrad probe has its intensity localized in its tails which results in a probe that expands towards the neighboring atomic columns (see Fig. 3). As a consequence, the maximum nonmagnetic signal intensity also shifts towards the nearest neighboring atomic columns for all thicknesses (see also Supplementary Fig. S4). In this case, the probed atomic column contains only 12% of maximum intensity of the nearest atomic columns.

Similar to the magnetic response, the maximum nonmagnetic signal for a vortex beam is strongly localized on the probed atomic column. Such response is maintained for all the calculated thicknesses. However, the localization on the probed atomic column is very strong for small thicknesses. For instance, at a thickness of 7.22 nm, the maximum signal intensity on the nearest neighbor column is only 2.7% of the intensity coming from the probed atomic column. Whereas for higher thicknesses, the nearest neighbor column contains up to 30% of the maximum intensity of probed atomic column. As pointed out above, a vortex beams also show non-negligible spreading as it propagates through the lattice.

## Atomic column resolved profiles of magnetic and non-magnetic signals

In analogy with radial profiles of energy filtered diffraction patterns shown in Fig. 5, in this section, we present the radial profiles of EMCD signal and non-magnetic signal, shown separately for individual groups of neighboring atomic columns, as marked in Fig. 1(a).

**Figure 8:**
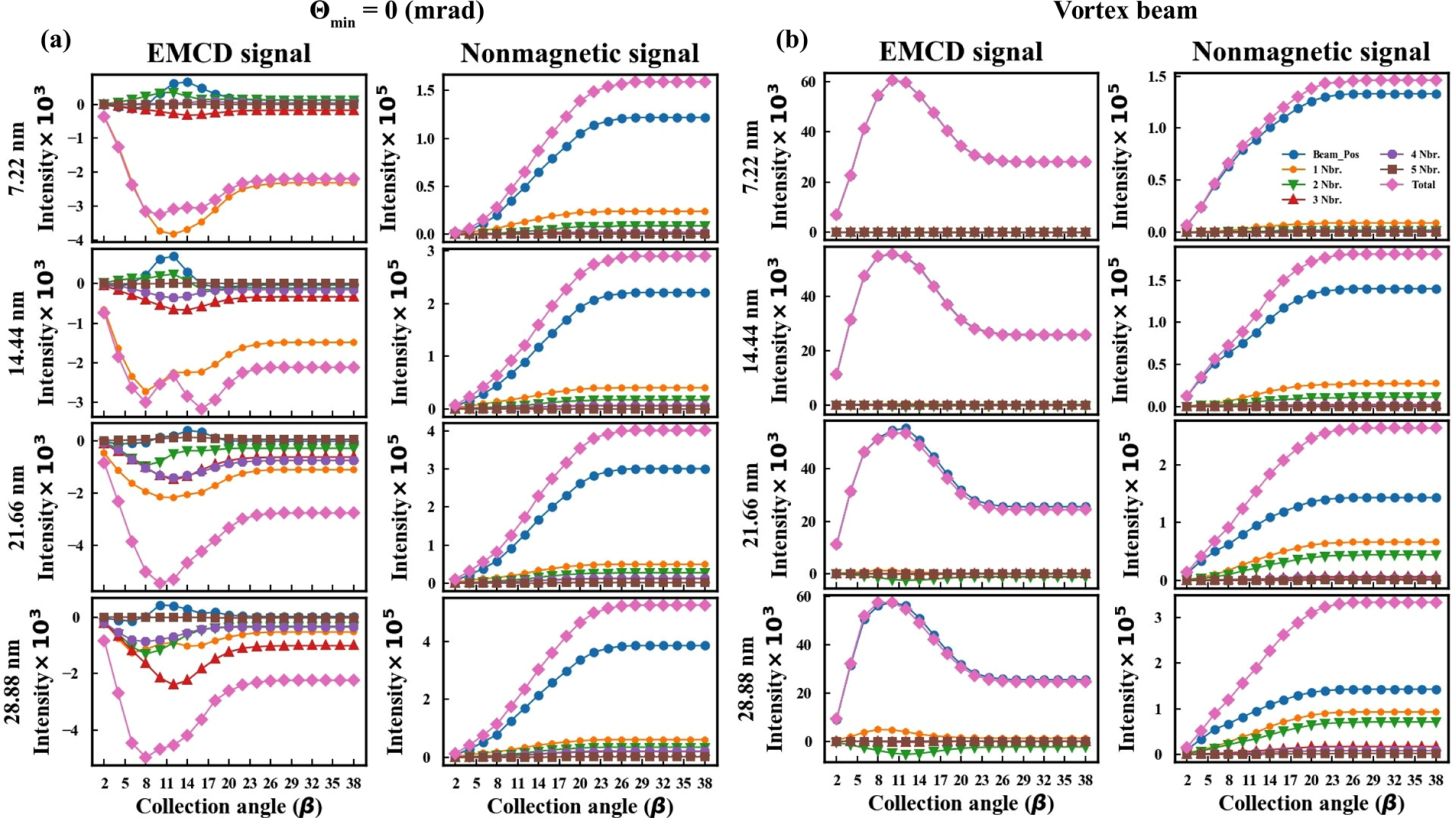
Radial profiles of atomic column contributions for (**a**) Θ_min_ = 0 astigmatic probe and (**b**) vortex beam.

Figure 8(a) shows the radial profiles for the Θ_min_ = 0 mrad astigmatic probe. For all thicknesses, the integrated signal is primarily contributed by the nearest neighbor atomic columns, while the probed atomic column remains the major source of the nonmagnetic signal. We see an efficient mutual cancellation of the positive and negative EMCD contributions from the probed atomic column. For Θ_min_ = 20 mrad probe, the major nonmagnetic signal contributor atomic column shifts towards the nearest neighbor atomic columns. We have seen such trend in the energy filtered diffraction patterns of the nonmagnetic signal for individual atomic columns.

Figure 8(b) shows the similar radial profiles calculated for the vortex beam. For a vortex beam, both the magnetic and nonmagnetic signal are dominated by the probed atomic column. This is also reflected in how strongly the vortex beam remains localized at the probed atomic column. Opposite to the aberrated beams, the magnetic and nonmagnetic signals are both strongly localized at the probed atomic column and steeply decay on the next neighboring atomic columns. Irrespective of increasing thickness, the vortex beam remains well localized on the probed atomic column. These results further reinforce the view that a vortex beam can be very effective probe to measure atomically localized magnetic properties. Note however, that while the nonmagnetic signal intensity grows with sample thickness, the EMCD signal changes very little from its values at about 14 nm. This suggests that vortex beam sensitively samples the magnetic properties of the top 10–15 nm of the sample, while it is less sensitive to magnetism arising deeper inside the sample. To gain further insight into this observation one needs to analyze the individual atomic contributions to the magnetic signal.

### Individual atomic contributions to the inelastic scattering cross-section

**Figure 9:**
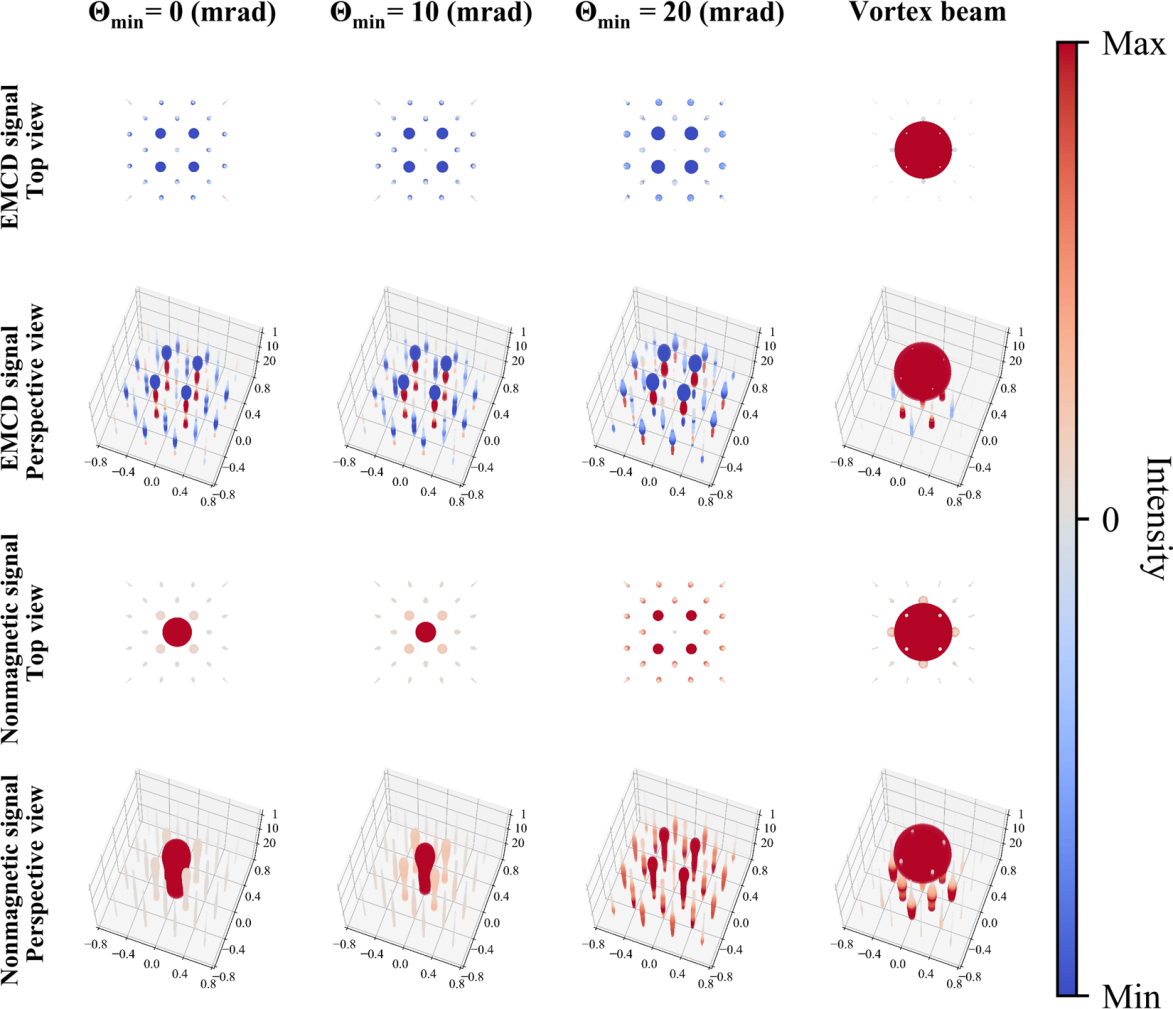
Two and three-dimensional views of individual atomic contributions to the magnetic and nonmagnetic signal. Each atom’s contribution is represented by a sphere of a radius proportional to its absolute value; (large) positive values are (dark) red, while negative values are blue.

Figure 9 represents the three dimensional projection and perspective profiling of magnetic and nonmagnetic signal, arising from individual atomic columns in the LaAsMnO crystal. As expected, for astigmatic probes we see magnetic signals being dominated by the nearest neighbor atomic columns. The perspective view for the EMCD signal indicates its often observed oscillating behavior as the thickness increases^43^.

As anticipated in the previous section, for an electron vortex beam, the major contribution to the magnetic signal arises within the first few nanometers of the sample. This explains the lack of increase of total EMCD in Fig. 8(b). Note also the growing negative contributions from the second nearest neighbor columns, as the thickness increases (cf. Fig. 6b).

In contrast to the EMCD signal, the nonmagnetic signal is mainly associated with the probed atomic column for all probes except for the Θ = 20 mrad astigmatic probe, where the nonmagnetic signal originates mostly from the nearest neighbor atomic columns. Generally one can observe shrinking of the nonmagnetic contributions from the probed atomic column, as the Θ_min_ is growing. For the vortex beam, the signal localization is strong, coming mostly from the probed column. Nevertheless, as the beam spreads, at larger depths in the crystal there are non-negligible contributions also from the neighboring columns.

## Conclusions

We have studied the localization of magnetic dichroic signal on atomic columns in EMCD, as observed with phase aberrated probes and a vortex beam. We show that by introducing an annular convergence aperture in the aberrated probes, the magnetic SNR can be enhanced. Vortex beams are predicted to be very efficient probes for atomic scale magnetometry. Optimization of the SNR constrains the signal detection within rather small collection angles of ~6–8 mrad. With four-fold astigmatic probes the magnetic signal originates mostly from the nearest neighbor atomic columns, while with a vortex beam the magnetic signal remain strongly localized within the probed atomic column. Considering the excellent localization and high SNR, we suggest that vortex beams can be used to study the crystal site specific magnetic properties, magnetism on material interfaces, magnetic property of doped impurities, etc. with atomic spatial resolution. We believe that the information presented here will help experimentalists to create road maps for future EMCD experiments with atomic size electron beams.

## Electronic supplementary material

Supplementary Information
