# Supplementary material for: Probing the localization of magnetic dichroism by atomic-size astigmatic and vortex electron beams
Source: Sci Rep. 2018 Mar 5;8:4019. doi: 10.1038/s41598-018-22234-8 (PMC5838113; doi:10.1038/s41598-018-22234-8)
Supplement: Supplementary file 1 — Supplementary Information [file 41598_2018_22234_MOESM1_ESM.pdf]

Supplementary information

Probing the localization of magnetic dichroism by atomic-size astigmatic and vortex electron beam.

Devendra Singh Negi,[1*] Juan Carlos Idrobo,[2] Ján Rusz[1]
[1]Uppsala University, Department of Physics and Astronomy, Uppsala, 75237, Sweden
[2] Oak Ridge National Laboratory, Center for Nanophase Materials Sciences, Oak Ridge, Tennessee, 37831, USA
[*]devendra.negi@physics.uu.se

1 Radial profiling of EMCD, Nonmagnetic, Relative EMCD, SNR for various probes at different thicknesses.

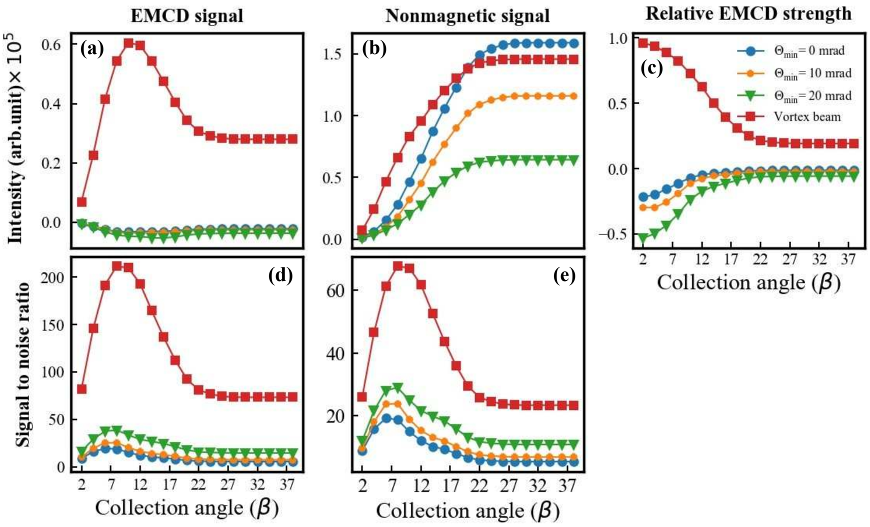

Figure S1: Radial profile of (a) Magnetic (b) Nonmagnetic (c) Relative magnetic signal (d) SNR (e) SNR normalized per unit of beam current prior to beam forming aperture, shown for various probes at 7.22 nm thickness.

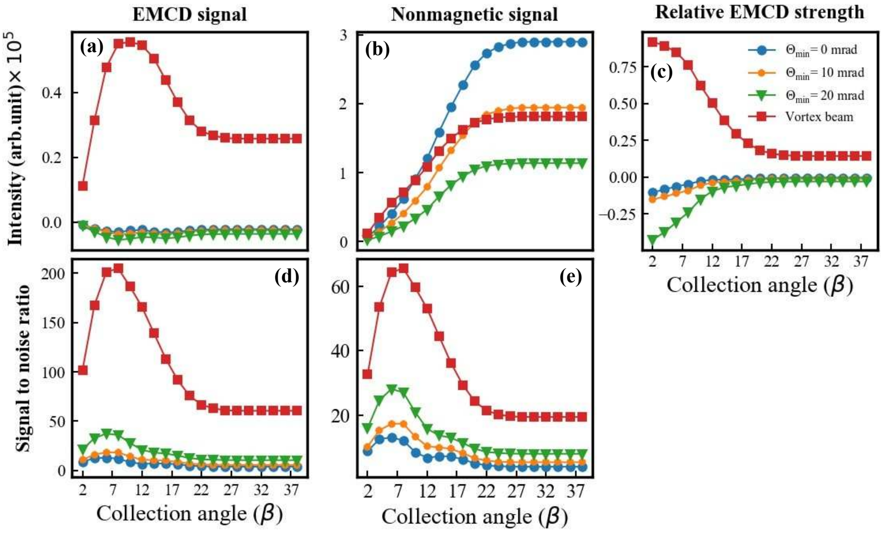

Figure S2: Radial profile of (a) Magnetic (b) Nonmagnetic (c) Relative magnetic signal (d) SNR (e) SNR normalized per unit of beam current prior to beam forming aperture, shown for various probes at 14.44 nm thickness.

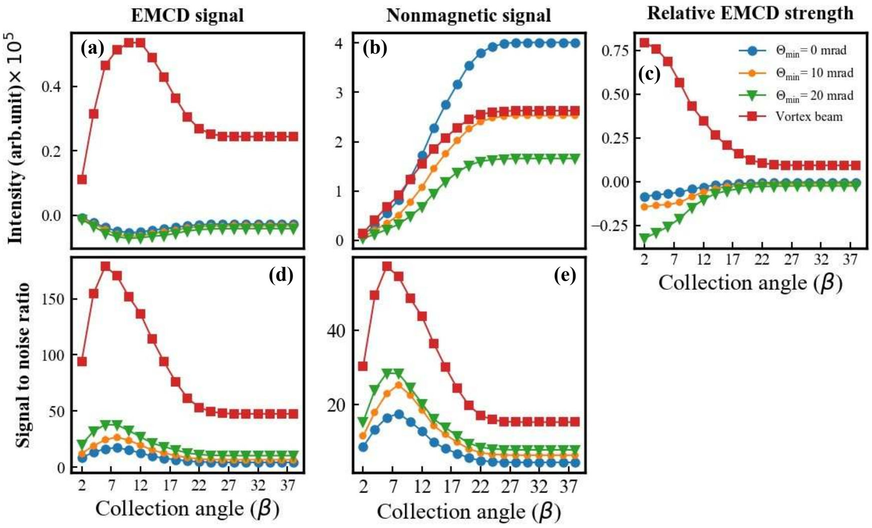

Figure S3: Radial profile of (a) Magnetic (b) Nonmagnetic (c) Relative magnetic signal (d) SNR (e) SNR normalized per unit of beam current prior to beam forming aperture, shown for various probes at 21.66 nm thickness.

Figures S1, S2, S3 show the radial profiling for EMCD, Nonmagnetic, Relative EMCD, SNR, as a function of collection angle. For all thicknesses (7.22 nm, 14.44 nm, 21.66 nm) vortex beam shows stronger EMCD signal and SNR within a narrow range of collection angles (6 - 8 mrad).

2 EMCD signal contribution from individual atomic columns.

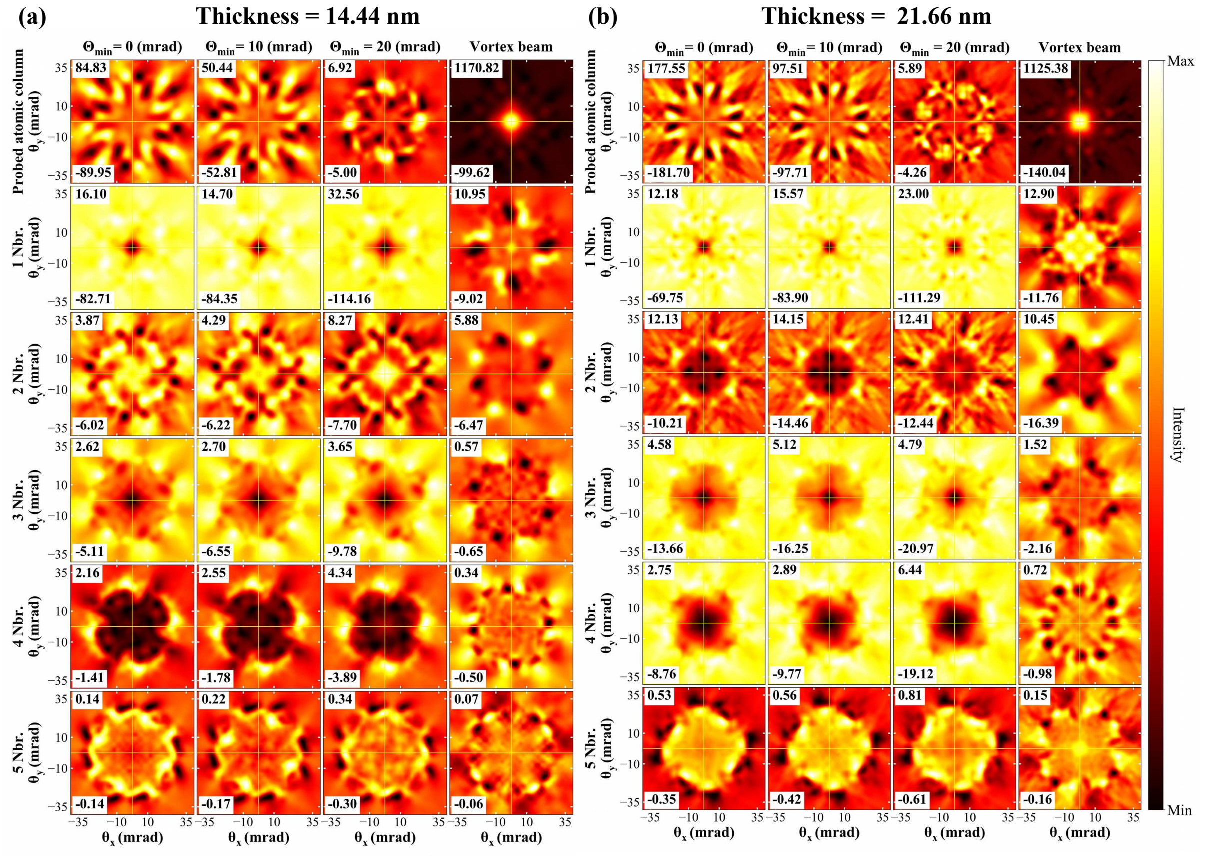

Figure S4: EMCD signal contribution from individual atomic columns originating from various probes, at 14.44 nm and 21.66 nm thicknesses.

3 Nonmagnetic signal contribution from individual atomic columns.

Figure S4 represents the EMCD signal contribution, arising from the individual atomic columns for two intermediate thicknesses of 14.44 nm and 21.66 nm, respectively. For astigmatic probes, the nearest neighbor atomic column remains the major source of EMCD signal. The EMCD signal decays on more distanced atomic columns. On other hand, while probing with vortex beam, probed atomic column remains the major source of EMCD signal. The signal is drastically reduced for other atomic columns. Figure S5 represent the nonmagnetic signal intensity, arising from the individual atomic columns. For vortex beam and astigmatic probes, Θ_{min}= 0, 10 mrad, the probed atomic column remains the major source of nonmagnetic signal. However, due to the higher spreading for the probe Θ_{min}= 20 mrad, the major source of the nonmagnetic signal intensity shifts towards the nearest neighbor atomic column.

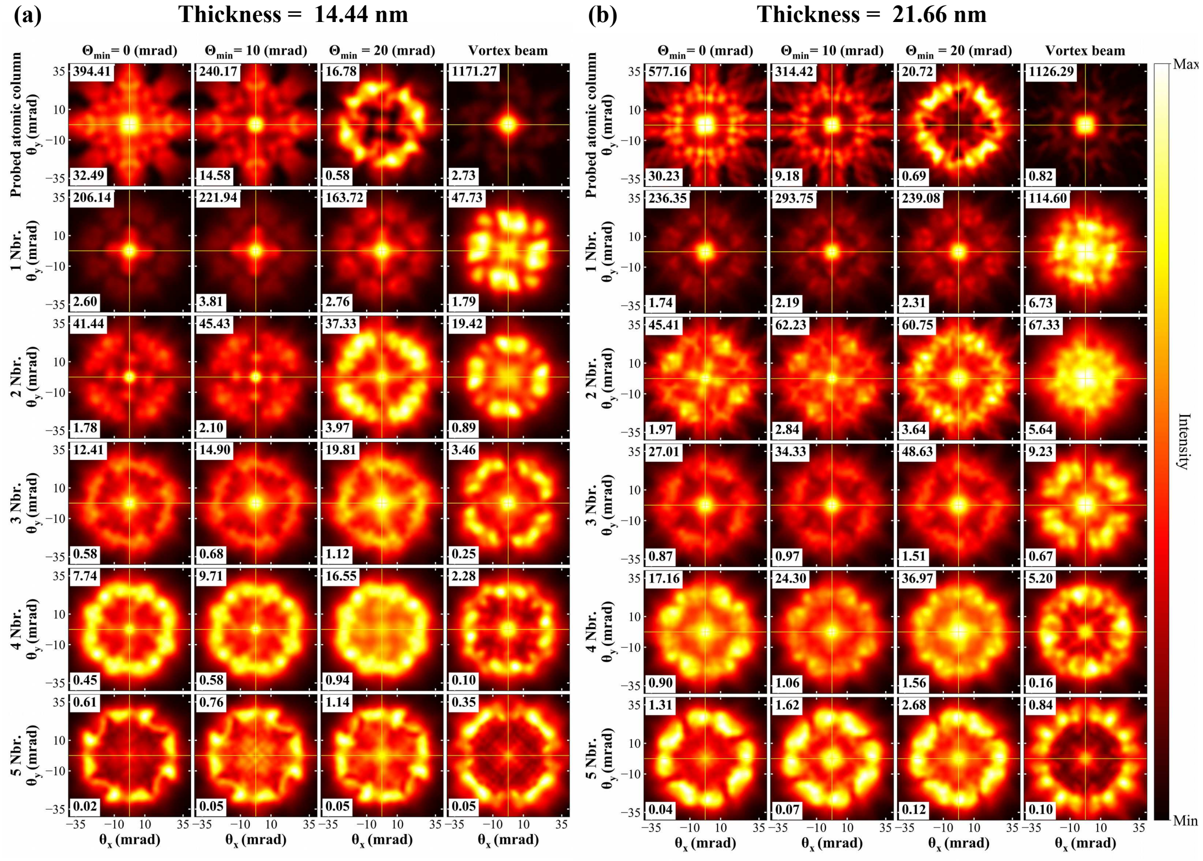

Figure S5: Nonmagnetic signal contribution from individual atomic columns originating from various probes at 14.44 nm and 21.66 nm thicknesses.

4 Radial and flux distribution of the probes.

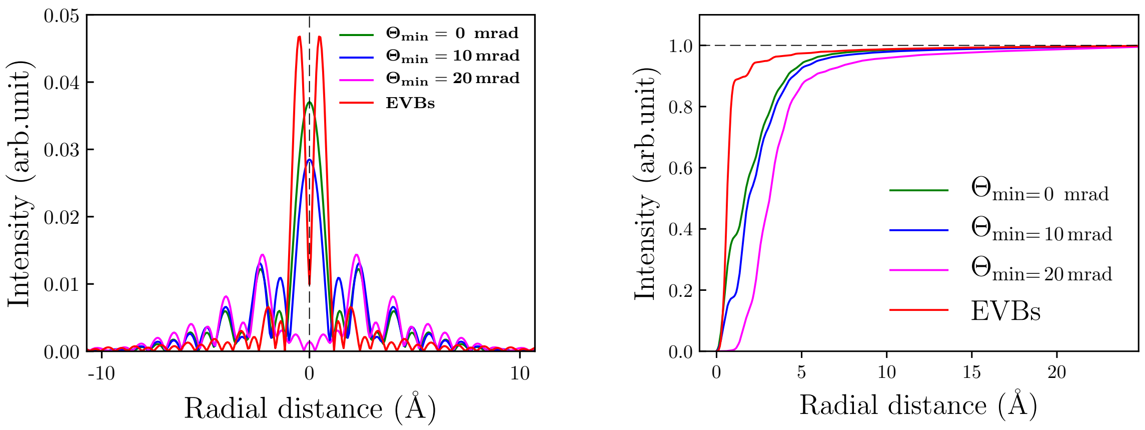

(a) (b)

Figure S6: (a) Intensity of the probes as function of radial distance. (b) Flux distribution of the probes as a function of radial distance.

In Figure S6 we qualitatively compare the flux distribution among the various probes used in the present investigation. Figure S6(a) represents the scan along the wave front of the probes. From Fig.S6(a), it can

be seen that for $\Theta_{\rm min} = 0, 10\,$mrad probe the maximum flux distribution lies on the center of the probe, whereas by introducing the aperture i.e, $\Theta_{\rm min} = 20\,$mrad the flux accumulates far from the center. However, as in the case of EVBs there exist the phase singularity at the center and major flux distribution lies far from the center. Figure S6(b) represents the flux distribution as a function of radial distance. From the Fig.S6(b), we estimate that 50% (or 90%) of the beam intensity is located within a radius of 1.68, 1.93, 3.15, 0.64 Å(or 4.18, 4.57, 5.73, 1.74 Å) for astigmatic probes and vortex beam, respectively. This measure has been used instead of usual full-width half-maximum (FWHM), because astigmatic probes are not rotationally symmetric and, especially, the $\Theta_{\rm min} = 20\,$mrad probe does not have a maximum in its central peak. One can see that for astigmatic probes a non-negligible fraction of beam flux is carried by the side-lobes, which pass through neighboring atomic columns, as is discussed in the manuscript.
